# Magnetic solid-phase extraction of trace cobalt(II) on iron oxide-hexagonal boron nitride in water and food samples

**DOI:** 10.55730/1300-0527.3782

**Published:** 2025-11-21

**Authors:** Mustafa SOYLAK, Ali Mohammednour ALI MOHAMMED, Furkan UZCAN

**Affiliations:** 1 Department of Chemistry, Faculty of Science, Erciyes University, Kayseri, Turkiye; 2 Technology Research and Application Center (TAUM), Erciyes University, Kayseri, Turkiye; 3 Turkish Academy of Sciences (TÜBA), Ankara, Turkiye; 4 Department of Chemistry, Faculty of Education, Kassala University, Kassala, Sudan

**Keywords:** Cobalt, iron oxide/hexagonal boron nitride, food, water

## Abstract

Determination of trace cobalt(II) (Co(II)) in real samples remains challenging because of its very low concentration and strong matrix interferences. In this study, a simple and green magnetic solid-phase extraction (mSPE) method was developed for the selective separation and preconcentration of Co(II) ions using a newly designed iron oxide/hexagonal boron nitride (IOhBN) nanocomposite. The material was synthesized through a one-step, surfactant-free coprecipitation process, combining the high surface area, stability, and layered structure of hBN with the magnetic and reactive features of Fe_3_O_2_ nanoparticles. This hybrid structure provided abundant active sites and rapid magnetic separability, enabling efficient extraction within 5 min using only 15 mg of sorbent. The optimized conditions were pH 7.0, eluent type and volume (3.0 mol L^−1^ of HNO_3_, 0.5 mL), and total extraction time of 5 min. The method exhibited reliable and environmentally efficient analytical performance with a limit of detection of 0.67 μg L^−1^, a relative standard deviation of 4.2%, and an enrichment factor of 20. Validation with certified reference materials (water, BCR 505; onion NCS ZC 73033; spinach leaves, NIST1570a) and successful applications to water and food samples confirmed its reliability. The results demonstrate that the proposed IOhBN-based mSPE method is a novel, rapid, and environmentally sustainable approach for ultra-trace Co determination in complex matrices.

## 1 Introduction

Heavy metals, distinguished by their high atomic mass and toxic properties, present significant dangers to environmental stability and human health [1,2]. Their extensive application across diverse industries, including mining, manufacturing, and agricultural practices, has led to their widespread release into natural ecosystems, contaminating water sources, soil, and the atmosphere [3,4]. Due to their resistance to biodegradation, heavy metals persist in the environment, accumulating over time and posing severe risks to ecological balance and human populations [5]. Prolonged exposure to these pollutants has been associated with a variety of serious health complications, such as cognitive impairment, growth and developmental issues, cancer, and damage to critical organs [6,7]. Given the profound implications of metal pollution, it is essential to adopt comprehensive measures to mitigate this issue [8]. This includes enforcing stricter regulatory frameworks to control industrial emissions and waste disposal, promoting the development and adoption of sustainable technologies, and enhancing public awareness campaigns to educate communities about the health risks posed by heavy metal exposure [6,9].

Cobalt (Co) is a challenging metallic element that is silvery-gray and ductile. It has distinct chemical properties that closely resemble those of iron (Fe) and nickel, among the contaminants in the environment [10]. The sole recognized biological function of Co is its role as a metallic constituent of vitamin B_12_. Conversely, other Co compounds have been shown to pose environmental and health hazards following prolonged exposure. The distinctive blue hue of several Co compounds has led to their employment as a coloring ingredient for glass, ceramics, and jewelry for thousands of years [11,12].

Beyond its direct human health effects, the environmental release of Co from anthropogenic sources, such as mining activities, battery manufacturing, and industrial effluents, poses a significant ecotoxicological threat. Although Co is an essential micronutrient for some organisms, elevated concentrations in aquatic systems can exhibit significant toxicity to fish, algae, and invertebrates, thereby disrupting the ecological balance [13]. Its persistence and potential for bioaccumulation in the food chain necessitate vigilant and routine monitoring of environmental matrices, particularly industrial wastewater and natural water sources. However, quantifying Co at these environmentally relevant, ultra-trace concentrations (μg L^−1^) is highly challenging due to strong interferences from complex sample matrices. Therefore, the development of rapid, highly sensitive, and selective monitoring tools, such as the method proposed herein, is of paramount importance for effective environmental risk assessment and the enforcement of regulatory standards.

Trace metal detection in environmental samples has been easier in recent years. Techniques such as inductively coupled plasma mass spectrometry (ICP-MS) [14], flame atomic absorption spectrometry (FAAS) [15,16], inductively coupled plasma optical emission spectrometry (ICP-OES) [17], high-resolution continuous source atomic absorption spectrometry (HR-CS-AAS) [18,19], and graphite furnace atomic absorption spectrometry (GF-AAS) [20] are frequently utilized for this objective. Even with the progress made in analytical methods designed to reduce media interferences and improve sensitivity and selectivity, it is frequently essential to perform sample pretreatment before analyzing trace metals. Accurate and precise measurements often necessitate separation and enrichment techniques, mainly because of possible interferences and the low concentrations of trace species [21]. Various methods, including liquid-liquid extraction [22], cloud point extraction [23], and solid-phase extraction (SPE) [24–26], are employed for this enrichment process. Magnetic SPE (mSPE) stands out due to its cost-effectiveness, simplicity, lack of centrifugation, speed, and ability to provide a higher enrichment factor (EF) along with more reproducible results [27–30].

Magnetic nanomaterials are renowned for their remarkable magnetic qualities, biocompatibility, and biosafety [31]. These potential applications of nanoparticles are manifold and extend to areas such as biology, medicine, and the preservation of the environment. These include drug delivery, gene therapy, magnetic resonance imaging, and cancer diagnosis and treatment [32]. Researchers are also investigating functionalized magnetic nanoparticles for multiple applications [33,34].

Rapid advancements in nanotechnology have made nanomaterials widely used in many different fields. As these materials are used more and more in everyday consumer goods, serious concerns about the possible harmful effects of nanoparticles on human health have surfaced. Nanostructured materials consist of particles at the nanometer scale, which can be organized in one, two, or three dimensions [35]. Boron nitride (BN) is an exceptionally versatile inorganic material that has attracted considerable attention due to its remarkable properties and wide-ranging applicability in advanced industrial sectors. Its unique characteristics, such as outstanding oxidation resistance, superior thermal conductivity, excellent electrical insulation, chemical stability, low friction coefficient, nontoxic nature, and ecofriendliness make it a material of choice for cutting-edge technologies. While BN can crystallize in multiple forms, including cubic, rhombohedral, wurtzite, and amorphous structures, the hexagonal phase is the most stable and prevalent. This phase exhibits a layered structure similar to graphite, making it particularly valuable for applications requiring thermal management, lubrication, and electrical insulation. Boron (B) and nitrogen (N) atoms in hexagonal BN (hBN) are interconnected via strong covalent sp^2^ bonds, forming hexagonal B_3_N_3_ rings. These 2D layers are then stacked vertically, bound by weak van der Waals interactions, facilitating the material’s unique mechanical and thermal properties [36,37].

This study aimed to design, synthesize, and evaluate a cutting-edge nanocomposite material composed of hBN sheets and iron oxide (Fe_3_O_4_, IO) nanoparticles for the efficient and sensitive extraction of Co from samples. The applicability of this approach was successfully demonstrated through the determination of trace Co concentrations in various samples, such as water, fish, coffee, and onions.

The novelty of the present study lies in the design and application of a new hybrid magnetic nanosorbent composed of IO anchored on hBN (IOhBN) for the first time in mSPE of Co(II). Unlike previously reported sorbents that often require multistep functionalization or surfactant-assisted synthesis, the IOhBN composite was prepared through a simple, one-step coprecipitation method without any toxic organic reagents. The unique combination of the high surface area and chemical stability of hBN with the magnetic separability of Fe_3_O_4_ nanoparticles resulted in a material exhibiting reliable and environmentally efficient adsorption performance and rapid magnetic recovery. Moreover, the proposed mSPE procedure required only 15 mg of sorbent, and completed the adsorption–desorption cycle within 5 min, making it a green, rapid, and cost-effective approach. The method demonstrates high sensitivity (limit of detection (LOD) = 0.67 μg L^−1^), good precision (relative standard deviation (RSD) = 4.2%), and an EF of 20, outperforming or matching most existing Co(II) extraction methods. Its successful validation with certified reference materials (CRMs) and diverse real samples (water and food matrices) further confirmed its robustness and practical applicability.

## 2 Experimental

### 2.1. Instrumentations and chemicals

Atomic absorption measurements were conducted using an HR-CS-FAAS ContrAA 800 spectrometer (Analytik Jena AG, Jena, Germany) equipped with a 300W xenon short arc lamp as the radiation source. A WTW 3110 digital pH meter (WTW GmbH, Weilheim, Germany) was utilized to measure the pH levels of the solutions. Precise weighing of compounds was achieved with a Radwag AS220/C/2 analytical balance (Radwag, Balances and Scales, Radom, Poland), offering a sensitivity of 0.1 mg, while centrifugation was performed using a Rotofix 32 A centrifuge (Hettich GmbH, Tuttlingen, Germany). A vortex mixer (WhirliMixer; Fisons Scientific Equipment, Loughborough, England) enhanced the interaction between the sorbent and analyte. Fourier-transform infrared (FT-IR) spectra were obtained using a FT-IR 400 spectrometer (Perkin-Elmer, Waltham, MA, USA), covering a spectral range of 4000 to 500 cm^−1^. Structural and elemental characterization of the adsorbent was performed using a Zeiss Gemini 500 field emission scanning electron microscope (FE-SEM) (Carl Zeiss NTS GmbH, Oberkochen, Baden-Württemberg, Germany) coupled with energy-dispersive X-ray (EDX) analysis and SEM. X-ray diffraction (XRD) patterns were acquired using a diffractometer equipped with a monochromatic lens. An Ethos Lean compact microwave system (Milestone, Bergamo, Italy) was utilized to digest actual samples.

All reagents and chemicals (obtained from Merck KGaA, Darmstadt, Germany) used in this research were of analytical grade. Double-distilled deionized water (18.2 MΩ cm; Millipore Corp., Burlington, MA, USA) was used. To prepare stock solutions of Co(II), a standard solution of 1000 mg L^−1^ Co(II) was diluted to 50 mL with ultrapure water, yielding an intermediate stock solution of 40 mg L^−1^ Co(II). The stock solutions of various ions (10 g L^−1^) were prepared using 99.9% grade reagents (Merck KGaA). Key reagents such as ethanol (C_2_H_5_OH, 96%), iron(II) sulfate heptahydrate (FeSO_4_·7H_2_O, ≥99%), ammonia solution (NH_4_OH, 25%), nitric acid (HNO_3_, ≥65%), and sodium hydroxide (NaOH, 97%) were also used (Merck KGaA), as was hBN (≥99%) (Nanografi Nanotechnology, Ankara, Türkiye). For pH adjustment, aqueous solutions were prepared using acetate buffer solutions (pH 4.0–5.0), phosphate buffer solutions (pH 3.0–6.0 and 6.0–8.0), and an ammonia buffer solution (pH 9.0). The preparation of these buffer solutions required sodium phosphate dibasic heptahydrate (Na_2_HPO_4_·7H_2_O), sodium phosphate monobasic monohydrate (NaH_2_PO_4_·H_2_O), sodium acetate (CH_3_COONa), and acetic acid (CH_3_COOH) (Merck KGaA).

### 2.2. Synthesis of IO@hBN (IOhBN)

IO nanoparticles were fabricated through a coprecipitation approach, following a well-documented procedure outlined in the literature [38]. The synthesis process involved the gradual and continuous addition of 50 mL of an aqueous ammonium hydroxide solution (1.0 mol L^−1^ of NH_4_OH) to an equal volume of an aqueous iron(II) sulfate heptahydrate solution (0.1 mol L^−1^ of FeSO_4_·7H_2_O). This reaction was conducted under ultrasonic conditions, and maintained at 50 °C to ensure uniform mixing and nucleation. Following this, the mixture was subjected to further heating in a water bath at 120 °C for 20 min to promote the complete formation and stabilization of the nanoparticles. The resulting particles were then isolated using an external magnetic field. The nanoparticles were washed three times with 0.01 mol L^−1^ of HCl under centrifugation to eliminate residual impurities. After purification, the nanoparticles were dried at 60 °C for 24 h to obtain a stable, dry powder. This synthesis protocol ensured the production of high-purity, well-defined IO nanoparticles suitable for various advanced applications [38].

**Figure 1:**
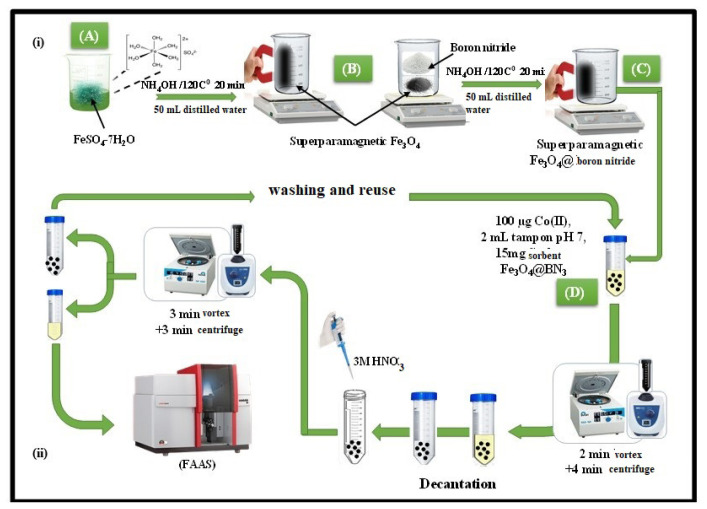
Schematic diagram of the IOhBN synthesis (a) and IOhBN/mSPE procedure (b).

IOhBN was synthesized using a coprecipitation method, as described in the literature, with the addition of hBN to the process [38, 39]. Subsequently, 0.5 g of hBN was dispersed in 50 mL of deionized water to form a stable suspension. This hBN suspension was then introduced into the synthesis process. The procedure involved the continuous addition of 50 mL of an aqueous NH_4_OH solution (1.0 mol L^−1^) to 50 mL of an aqueous FeSO_4_·7H_2_O solution (0.1 mol L^−1^) under ultrasonic conditions at 50 °C, in the presence of the hBN suspension. The mixture was subsequently heated in a water bath at 120 °C for 20 min to facilitate the formation of nanoparticles. The resulting particles, now integrated with hBN, were separated using an external magnetic field and washed three times with 0.01 mol L^−1^ of HCl under centrifugation to remove impurities. Finally, the purified nanoparticles were dried at 60 °C for 24 h to obtain the final composite material. This modified method ensured the production of a hybrid material (Figure 1a) [39].

### 2.3. IOhBN/mSPE procedure

A sample solution was prepared in a 50-mL centrifuge tube by mixing 2 μg of Co(II), corresponding to a concentration of 40 ng mL^−11^, with 2 mL of phosphate buffer solution adjusted to a pH of 7.0. Subsequently, 15 mg of the IOhBN composite adsorbent was introduced to this solution. The mixture was then vigorously vortexed for 2 min to ensure optimal contact and interaction between the Co(II) ions and the composite material. After the adsorption process, an external neodymium magnet was employed to facilitate the rapid and efficient separation of the solid phase composite material with adsorbed Co(II) from the liquid phase. This allowed effectively isolation of Co(II) from the solution. To recover the adsorbed Co(II) ions, the composite was treated with 0.5 mL of 3M HNO_3_ and vortexed for 3 min. The Co(II) concentration in the eluent was then accurately determined using FAAS. This procedure demonstrates a highly effective and reliable method for the extraction, preconcentration, and quantification of trace amounts of Co(II) in aqueous samples, utilizing the unique magnetic and adsorptive properties of the IOhBN nanocomposite (Figure 1b).

### 2.4. Applications

Water samples were obtained from regions across Türkiye, with wastewater collected explicitly from the Kayseri-Industrial Zone. The samples were analyzed directly without any prior filtration. The IOhBN/mSPE method was applied to these samples using the standard addition approach to ensure precise and dependable quantification of the target analytes. Each sample, including canned tuna, onion, spinach, and coffee, was homogenized for consistency and dried in an oven. Exactly 1.0 g of each homogenized sample was measured and placed into separate beakers. To initiate digestion, 10 mL of HNO_3_ was added to the samples, and then the mixtures were heated at 80 °C until near dryness. After cooling, 5 mL of concentrated HNO_3_ and 5 mL of H_2_O_2_ were added to the residues, and the mixtures were then heated again to ensure complete oxidation of the organic matter. After complete digestion, the samples were cooled, and deionized water was added to the residues to achieve a final volume of 20 mL. The pH of each solution was adjusted to approximately 7.0 by dropwise addition of 10 M sodium hydroxide under continual stirring, using a calibrated pH meter. The solutions were then transferred into 50-mL centrifuge tubes, and the IOhBN/mSPE was applied for further extraction and preconcentration of the target analytes [40].

For the CRMs, the IOhBN/mSPE method was directly applied to estuarine water (BCR 505) without pretreatment. For onion (NCS ZC 73033) and spinach leaves (NIST 1570a), 0.5 g of each CRM was weighed, and the same digestion procedure described above was meticulously followed to ensure consistency and accuracy. This comprehensive and methodical approach highlights the versatility and robustness of the IOhBN/mSPE method for analyzing a wide range of samples, including environmental water, wastewater, and complex food matrices, while ensuring high selectivity and sensitivity.

## 3 Results and discussion

### 3.1. Characterization of the IOhBN

The XRD analysis provided critical insights into the crystalline structure and phase composition of the IOhBN nanocomposite, confirming the successful integration of IO and hBN. As illustrated in Figure 2A(a), the XRD pattern of IO exhibited distinct diffraction peaks at 2θ values of 30.2°, 35.5°, 43.0°, 57.0°, and 62.5°. These peaks were indexed to the crystallographic planes (220), (311), (400), (511), and (440), respectively, and are consistent with the standard reference data (JCPDS card no. 19-0629). These well-defined peaks indicate the crystalline nature of the IO component within the nanocomposite [41]. In addition, the XRD pattern of hBN, depicted in Figure 2A(b), revealed a prominent and sharp peak at 26.8°, corresponding to the (002) crystallographic plane of hBN. This peak is characteristic of the hexagonal graphitic-like structure of BN, typically observed at 2θ ≈ 26°. Additionally, weaker diffraction peaks were observed at 41.7°, 43.95°, 50.25°, and 55.1°, which were attributed to the (100), (101), (102), and (004) planes, respectively, and are consistent with JCPDS card no. 34-0421. These features further corroborate the hexagonal crystalline structure of BN [36].

The XRD results of the IOhBN nanocomposite, presented in Figure 2A(c), demonstrated a combination of the diffraction peaks observed in both IO and hBN. The nanocomposite retained all the characteristic peaks of IO and hBN, confirming the coexistence of both materials within the composite structure. This overlapping of peaks from IO and hBN provided strong evidence of the successful synthesis of the IOBN nanocomposite. The preservation of the individual crystalline features of IO and BN in the composite suggests that the integration process did not disrupt their intrinsic structural properties, thereby validating the effectiveness of the synthesis methodology [36,41].

Figures 2B(a), 2B(b), and 2B(c) present the FT-IR spectra of Fe_3_O_4_, hBN, and the Fe_3_O_4_@hBN nanocomposite, respectively, within the wavenumber range of 4000–500 cm^−1^. The spectra displayed a broad absorption band at 3245 cm^−1^ associated with O–H stretching vibrations of adsorbed water molecules and surface hydroxyl groups. Fe–O vibrations were observed at approximately 575 cm^−1^ in both Fe_3_O_4_ and Fe_3_O_4_@hBN, while the characteristic B–N stretching vibration of hBN appeared near 895 cm^−1^ in both hBN and Fe_3_O_4_@hBN, confirming the structural integrity of hBN after composite formation. The absence of any new bands or major shifts in the Fe–O or B–N regions suggests that the interaction between Fe_3_O_4_ and hBN was mainly physical, most likely due to electrostatic attraction and surface adhesion, rather than the formation of new chemical bonds. These findings indicated that Fe_3_O_4_ nanoparticles were successfully anchored on the hBN surface while retaining the individual structural features of both components.

The FE-SEM images revealed distinct morphological differences among the IO (Figure 2C(a)), hBN (Figure 2C(b)), and IOhBN materials (Figure 2C(c)). The IO particles exhibited a coarse and irregular distribution, while the hBN nanoparticles exhibited a morphology characterized by a plate-like structure and a porous and rough texture. This unique morphology of hBN suggests a high surface area, which can significantly enhance its reactivity, making it particularly suitable for applications such as adsorption. In the case of the IOhBN nanocomposite, smaller IO particles were dispersed on the hBN structure, indicating successful integration of the two materials.

Further insights were provided by the SEM images of the IO (Figure 2C(d)), hBN (Figure 2C (e)), and IOhBN (Figure 2C(f)). The IO nanoparticles appeared as dot-like structures with an average size of approximately 10 nm. At the same time, the hBN maintained its leaf-like or plate-like morphology, which is consistent with the FE-SEM images. The SEM image of the IOhBN nanocomposite clearly shows IO particles uniformly distributed across the hBN surface, confirming the effective combination of the two components. Both imaging techniques suggest that the presence of IO plays a significant role in modifying the surface properties of the hBN nanoparticles. IO likely formed a coating around the hBN core, resulting in a denser and more compact surface structure. This morphological transformation, driven by the incorporation of IO, enhanced the suitability of the IOhBN nanocomposite for magnetic separation applications. The altered surface characteristics, combined with the unique structural properties of the nanocomposite, demonstrate its potential for advanced functional applications in materials science and engineering [42].

The FE-SEM-EDX analysis revealed notable changes in the chemical composition and surface morphology of the IOhBN nanocomposite compared to its components, IO and hBN. In the case of pure IO nanoparticles (Figure 2D(a)), Fe was the predominant element, constituting approximately 55% by weight, while oxygen (O) accounted for roughly 41% by weight. This composition aligns with the expected chemical structure of pure IO. The hBN nanomaterial (Figure 2D(b)) exhibited an almost equal distribution of B and N, with B at ~48% by weight and N at ~52% by weight, consistent with the stoichiometric composition of hBN. In contrast, the IOhBN nanocomposite (Figure 2D(c)) demonstrated a more complex and diverse elemental composition. B remained the dominant element at ~37% by weight, but significant contributions from O (~33% by weight), Fe (~20% by weight), and N (~20% by weight) were also observed. Incorporating IO into the nanocomposite resulted in a compact morphological structure and a notable increase in the Fe and O content. The elevated Fe content suggests the presence of IO within the nanocomposite, indicating that the IOhBN material was not merely a physical mixture but a chemically integrated system. The EDX results further support the formation of a core-shell structure, where hBN acted as the core, and Fe_3_O_4_ formed a coating around it. This core-shell configuration is critical for enhancing the functional properties of the nanocomposite, particularly for applications requiring magnetic responsiveness, such as magnetic separation. The successful integration of IO and hBN, along with the formation of Fe_3_O_4_, underscores the tailored design of the IOhBN nanocomposite for advanced material applications.

### 3.2. Effects of pH

In mSPE studies, the pH of both model and sample solutions plays a role in extraction the efficiency of target metal ion recovery on the adsorbent material. This parameter significantly influences several key processes, including the attraction of metal ions to the adsorbent surface, their elution—the equilibrium between the adsorbent and adsorbate—and the potential for metal hydroxide precipitation. For instance, the interaction between Co(II) ions and the IOhBN nanocomposite involves chemical reactions or bonding mechanisms that facilitate the adsorption or binding of metal ions onto the nanocomposite surface [43].

**Figure 3:**
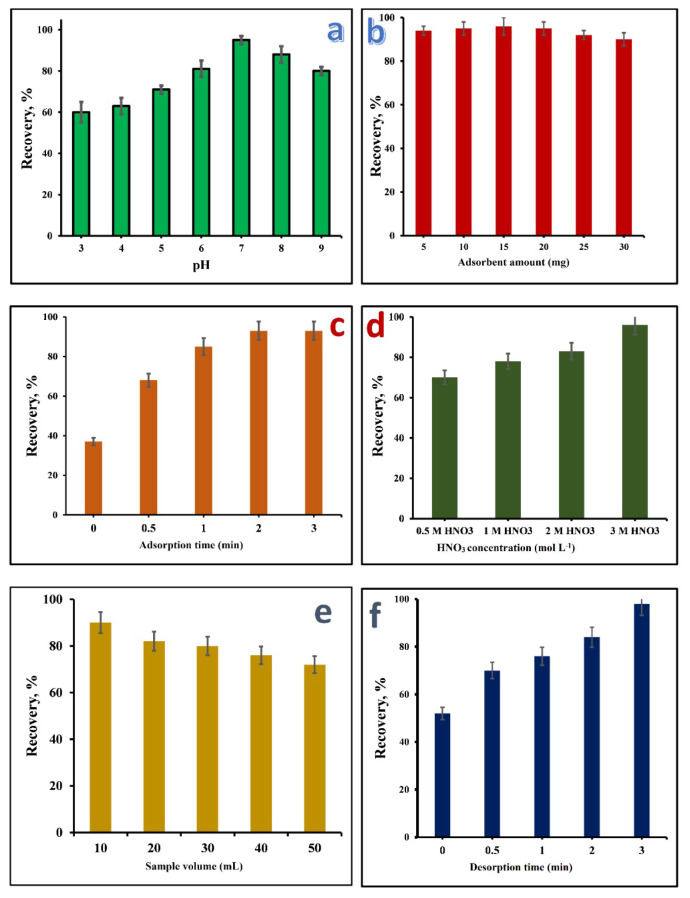
(a) Effect of pH on Co(II) recovery. Conditions: 40 μg L^−1^ of Co(II), 2 mL of buffer (pH 3.0–9.0), 30 mg of adsorbent, 3 min of adsorption/desorption, 10-mL sample volume, 5 mL of eluent (3.0 mol L^−1^ of HNO_3_). (b) Effect of the adsorbent amount on Co(II) recovery. Conditions: 40 μg L^−1^ of Co(II), pH 7.0, 5–30 mg of adsorbent, 3 min of adsorption/desorption, 10-mL sample volume, 5 mL of eluent (3.0 mol L^−1^ of HNO_3_). (c): Effect of the adsorption time on Co(II) recovery. Conditions: 40 μg L^−1^ of Co(II), pH 7.0, 15 mg of adsorbent, 0–3 min of adsorption, 3 min of desorption, 10-mL sample volume, 5 mL of eluent (3.0 mol L^−1^ HNO_3_). (d) Effect of the desorption time on Co(II) recovery. Conditions: 40 μg L^−1^ of Co(II), pH 7.0, 15 mg of adsorbent, 2 min of adsorption, 0–3 min of desorption, 10-mL sample volume, 0.5 mL of eluent (3.0 mol L^−1^ of HNO_3_). (e) Effect of the eluent concentration on Co(II) recovery. Conditions: 40 μg L^−1^ of Co(II), pH 7.0, 15 mg of adsorbent, 2 min of adsorption, 3 min of desorption, 10-mL sample volume, 5 mL of eluent (0.5–3.0 mol L^−1^ of HNO_3_). (f) Effect of the sample volume on Co(II) recovery. Conditions: 40 μg L^−1^ of Co(II), pH 7.0, 15 mg of adsorbent, 2 min of adsorption, 3 min of desorption, 10–50-mL sample volume, 0.5 mL of eluent (3.0 mol L^−1^ of HNO_3_).

The optimization of pH for Co(II) recovery revealed a distinct trend in the relationship between pH and recovery efficiency. At lower pH values (pH 3.0 and 4.0), the recovery percentages of Co(II) were relatively low, indicating that highly acidic conditions are not conducive to efficient Co extraction. This can be attributed to the positively charged surface of the IOhBN nanocomposite under acidic conditions, which repeled the positively charged Co(II) ions, thereby hindering effective adsorption. As the pH increased, a notable improvement in recovery was observed, with the highest efficiency achieved at pH 7.0 (Figure 3a). This suggests that slightly acidic to neutral conditions are optimal for Co(II) extraction. However, as the pH continued to rise beyond 7.0, recoveries began to decrease, likely due to the formation of Co(II) hydroxide species, which reduced the availability of free Co(II) ions for adsorption. Based on these findings, a pH of 7.0 was selected for subsequent experiments, as it strikes a balance between maximizing Co(II) recovery and minimizing the risk of hydroxide precipitation. This pH ensures efficient interaction between the Co(II) ions and the IOhBN nanocomposite while maintaining the system’s stability. The results underscore the importance of pH optimization in mSPE processes and highlight the tailored design of the IOhBN nanocomposite for practical metal ion extraction applications.

### 3.3. Adsorbent amount

Another critical parameter influencing the quantitative recovery of analytes in mSPE studies is the amount of adsorbent used [44]. The recovery efficiency of Co(II) ions is highly dependent on the dosage of the adsorbent material. To investigate the effect of the IOhBN adsorbent dose, preconcentration experiments were conducted using varying amounts of the adsorbent, ranging from 5 to 30 mg, as illustrated in Figure 3b. The results demonstrated that quantitative recovery of Co(II) ions was achieved with 15 mg of the IOhBN adsorbent. This optimal dosage highlights the material’s high porosity and extensive surface area, facilitating the effective adsorption of heavy metal ions. Interestingly, as the adsorbent increased beyond 15 mg, the recovery efficiency decreased, falling below 90%. This reduction in recovery at higher adsorbent doses may be attributed to factors such as particle aggregation or reduced accessibility of active sites due to overcrowding. Therefore, 15 mg was identified as the optimal adsorbent dosage for subsequent experiments, ensuring maximum recovery efficiency while minimizing material usage. Furthermore, the reusability of the IOhBN adsorbent was evaluated, and it was found that the material could be reused more than four times without any significant loss in its adsorption efficiency. This reusability underscores the durability and cost-effectiveness of the IOhBN nanocomposite, making it a promising candidate for sustainable and efficient mSPE applications in heavy metal ion extraction.

### 3.4. Adsorption and desorption durations

Adsorption and desorption times are critical factors that significantly influence the recovery efficiency of analytes in mSPE processes. To evaluate their impact, experiments were conducted using a vortex mixer with varying intervals ranging from 10 s to 3 min. The results, as depicted in Figure 3c for adsorption and Figure 3d for desorption, demonstrated a clear correlation between time and recovery efficiency. For adsorption, the recovery rate of Co(II) ions increased with longer contact times. This is attributed to the fact that extended adsorption durations allow more Co(II) ions to interact with and bind to the active sites on the surface of the IOhBN adsorbent. Similarly, during desorption, the recovery rate improved with increasing time as more Co(II) ions were eluted from the adsorbent surface into the eluent solution. This behavior demonstrated the importance of sufficient contact time for adsorption and desorption processes to achieve optimal recovery. The experimental data determined the optimal adsorption and desorption times as 2 and 3 min, respectively. These times ensure efficient adsorption of Co(II) ions onto the IOhBN adsorbent and their subsequent elution into the solution. The adsorption process is facilitated by the high affinity of the synthesized sorbent for Co(II) ions. In contrast, the desorption process depends on factors such as the type and concentration of the desorption solvent, as well as the contact time. The selected optimal time balance maximizes recovery efficiency and processing time, making the IOhBN nanocomposite a practical and effective adsorbent for mSPE applications.

### 3.5. Optimization of eluent concentration

The concentration of the eluent plays a pivotal role in determining the efficiency of analyte desorption in mSPE processes. It directly affects the interaction between the sorbent and the target, thereby impacting the recovery results. Identifying the optimum eluent concentration is essential and depends on several factors, including the eluent’s nature and the IOhBN adsorbent’s characteristics. HNO_3_ was tested at various concentrations to evaluate its effectiveness in desorbing Co(II) ions from the IOhBN nanocomposite. As illustrated in Figure 3e, the recoveries of Co(II) exhibited a strong dependence on the eluent concentration. Quantitative recovery was achieved at an HNO_3_ concentration of 3 mol L^−1^, indicating that this concentration provides the optimal balance between effective desorption and minimal interference with the adsorbent’s structure. A clear negative correlation was observed between the eluent concentration and recovery efficiency; as the concentration of HNO_3_ decreased, the recovery of Co(II) ions also decreased. This trend underscores the importance of using a sufficiently concentrated eluent to overcome the binding forces between the Co(II) ions and the adsorbent surface. Based on these findings, 3 mol L^−1^ of HNO_3_ was selected as the optimal eluent concentration. This optimized eluent concentration enhances the desorption process and highlights the practical applicability of the IOhBN adsorbent in mSPE workflows for heavy metal ion extraction.

### 3.6. Effects of the sample volume

The sample volume is a critical parameter in mSPE as it directly impacts the EF, which is essential for achieving high sensitivity and minimizing waste in analytical measurements [45]. Optimizing both the initial sample volume and the final eluent volume is crucial to maximize the EF and ensure efficient recovery of target analytes. In this study, the effect of the sample volume on Co(II) recovery was investigated using volumes (10–50 mL) in centrifuge tubes, as shown in Figure 3f. Quantitative recovery (≥ 90%) was achieved only at a volume of 10 mL. The recovery efficiency decreased significantly as the sample volume increased beyond 10 mL. This reduction in recovery at higher volumes can be attributed to diffusion limits or inadequate mixing, which hinder equidistributive contact between the IOhBN surface and Co(II) ions. In larger volumes, a 50-mL centrifuge tube may restrict effective interaction, leading to incomplete adsorption of the target ions.

In addition to the sample volume, the eluent volume was optimized. A final eluent volume of 0.5 mL was optimal for achieving high recovery efficiency. The EF, defined as the ratio of the initial sample volume to the final eluent volume, was calculated as 20 times. This high EF underscores the effectiveness of the IOhBN adsorbent in concentrating Co(II) ions from dilute solutions, making it a promising material for trace metal analysis. These findings highlight the importance of optimizing sample and eluent volumes in mSPE workflows to achieve high recovery efficiency and sensitivity while minimizing resource consumption. Using a 10-mL sample and 0.5-mL eluent volume ensures practical applicability and analytical performance in Co(II) extraction studies.

### 3.7. Matrix effects

The influence of potentially interfering matrix ions on the extraction efficiency was systematically investigated to evaluate the selectivity of the developed IOhBN/mSPE method for Co determination. The tolerance limit, defined as the maximum concentration of interfering ions that causes no more than a ±5% deviation in the recovery of the target analyte (Co(II)), was determined for various cations and anions. These included Na^+^, K^+^, Mg^2+^, Ni^2+^, Cu^2+^, Cr^3+^, Pb^2+^, NO_3_−, Cl-, SO_4_^2−^, and CO_3_^2−^, which were individually added to model solutions to assess their impact on Co(II) recovery. As summarized in Table 1, the tolerance limits for these interfering species ranged from 5 to 2500 mg L^−1^. The results indicate that most foreign ions remain in the liquid phase during extraction, exhibiting negligible interference with Co(II) recovery. Notably, transition metal ions such as Ni^2+^, Cu^2+^, Zn^2+^, Cr^3+^, and Fe^3+^, even at concentrations 125–500 times higher than that of Co(II) (40 μg L^−1^), did not significantly affect the analysis. This demonstrates the high selectivity of the IOhBN adsorbent for Co(II) ions in the presence of competing metal ions. However, some exceptions were observed. Ni^2+^, while showing minimal interference at lower concentrations, may exert a more significant influence at higher levels. Additionally, Cr^3+^ and NO_3_^−^ were found to slightly affect the recovery of Co(II), though their impact remained within acceptable limits under the tested conditions. These findings highlight the robustness of the IOhBN/mSPE method for Co(II) determination, even in complex matrices containing high concentrations of potentially interfering ions. The high selectivity and tolerance limits of the IOhBN adsorbent make it a promising material for precise and accurate Co(II) determination in environmental and industrial samples where diverse interfering species are common.

### 3.8. Analytical performance

The developed IOhBN/mSPE approach exhibited remarkable sensitivity for the detection of Co(II), achieving an LOD as low as 0.67 μg L^−1^ and a limit of quantification (LOQ) of 2.23 μg L^−1^. These exceptionally low thresholds allowed for reliable Co(II) identification at ultra-trace concentrations. The LOD and LOQ were determined using the formulas LOD = (3 × σ) / m and LOQ = (10 × σ) / m, where σ denotes the standard deviation of absorbance measurements from 10 blank solutions, and m represents the slope of the calibration curve following the preconcentration step. The method also showcased high precision, with an RSD of just 4.2%. Strong linearity was observed between Co(II) concentrations and the instrument’s response, as evidenced by a high coefficient of determination (R = 0.9943) and the linear equation y = 2.1682x – 0.0136. This confirmed the method’s reliability across a broad concentration range. An EF of 20 highlights the method’s effectiveness in separating and preconcentrating Co(II) from complex matrices. These findings underscore the method’s capability to accurately and reproducibly isolate and enrich Co(II) from diverse sample types, with a broad linear detection range of 10–500 μg L^−1^.

The analytical performance of the method was validated using three CRMs: estuarine water, onion, and spinach leaves (Table 2). The certified Co(II) values of these materials were 0.99 nmol kg^−1^, 0.59 μg/g, and 0.393 μg/g, respectively. The measured values aligned closely, with 1.04 nmol kg^−1^ for estuarine water, 0.59 μg/g for onion, and 0.365 μg/g for spinach leaves, yielding recovery rates of 105%, 92%, and 93%, respectively. These recovery percentages, within the acceptable range of 90%–110% for trace analysis, demonstrate the method’s accuracy and reliability. This validation confirms the suitability of the IOhBN/mSPE technique for precise and accurate Co(II) quantification in complex sample matrices.

### 3.9. Applications

Table 3 summarizes the recovery values obtained for Co(II) after applying the IOhBN/mSPE technique to various food samples, including onion, coffee, tuna fish, and spinach. In the nonspike control samples, the natural levels of Co(II) were relatively low, with concentrations of 0.71 μg/g in spinach, 0.36 μg/g in onion, 0.43 μg/g in coffee, and 0.029 μg/g in tuna fish. To evaluate the method’s performance, recovery studies were conducted by spiking the samples with Co(II) at concentrations of 1 and 2.5 μg/g, except for tuna fish, which was spiked at lower levels of 0.05 and 0.1 μg/g due to its inherently lower Co(II) content. The recovery results demonstrated the method’s high efficacy in accurately extracting and quantifying Co(II) across various concentrations. Recovery rates ranged between 95% and 108%, indicating excellent accuracy. Notably, spinach and coffee consistently exhibited recovery values exceeding 100% at all the spiked concentrations, suggesting minimal matrix interference and high extraction efficiency. Furthermore, the method’s precision was confirmed by the observed low RSDs, with most samples showing accuracy levels below 5%. These findings underscore the reliability and robustness of the IOhBN/mSPE technique for determining Co(II) in complex food matrices, making it a valuable tool for food safety and quality control applications.

Table 4 presents the recovery results for wastewater and water samples spiked with varying concentrations of Co(II), highlighting the effectiveness of the IOhBN/mSPE method. Except for the tap water samples, all the unspiked samples exhibited detectable levels of Co(II), indicating the presence of this analyte in the tested matrices. Specifically, wastewater-1 and wastewater-2 samples showed baseline Co(II) concentrations of 92 and 81 μg L^−1^, respectively, suggesting some environmental contamination. The recovery rates for the spiked samples ranged from 90% to 108%, demonstrating the method’s high efficiency in extracting and preconcentrating Co(II) across a wide range of concentrations. These results underscore the robustness and reliability of the IOhBN/mSPE technique for accurately quantifying Co(II) in complex environmental samples, such as wastewater and natural water sources. The method’s ability to achieve consistent and precise recoveries, even in potential matrix interferences, highlights its suitability for environmental monitoring and contamination assessment applications.

### 3.10. Comparison of IOhBN/mSPE with other methods in the literature

This study evaluated the innovative IOhBN/mSPE method compared to previously reported techniques for extracting and quantifying Co(II) ions in various matrices. As illustrated in Table 5, the proposed method achieved competitive LODs relative to other methods documented in the literature. One of the key advantages of this approach is the simplicity of sorbent synthesis, which is more straightforward than other methods. Additionally, the proposed method requires only 15 mg of sorbent, making it more environmentally friendly and cost-effective. The IOhBN/mSPE technique demonstrates reliable and environmentally efficient analytical performance, including high sensitivity and precision. Furthermore, the magnetic properties of IOhBN simplify and expedite the extraction process, eliminating the need for additional equipment during the absorption and desorption stages. This not only enhances extraction efficiency but also reduces operational complexity. The proposed method offers several significant advantages, including simplicity, using environmentally benign solvents, rapid analysis times, low cost, ease of accessibility, and exceptional sensitivity. These features make the IOhBN/mSPE technique a desirable option for extracting and quantifying Co ions in various sample types, positioning it as a promising alternative to conventional methods in both research and practical applications.

The superior performance of the IOhBN composite compared to conventional Fe_3_O_4_-based or BN-supported sorbents originates from the synergistic combination of both components. In typical Fe_3_O_4_ nanocomposites, although magnetic separation is efficient, the particles tend to aggregate and show limited chemical stability and surface area, which can restrict adsorption performance. In contrast, the incorporation of hBN provides a chemically inert and thermally stable two-dimensional platform with a high specific surface area and abundant B–N active sites capable of coordinating metal ions. When Fe_3_O_4_ nanoparticles are anchored on the hBN surface, the resulting hybrid structure simultaneously exhibits reliable and environmentally efficient magnetic responsiveness, improved dispersion, enhanced adsorption capacity, and greater mechanical robustness. This synergy leads to faster separation, higher Co(II) recovery efficiency, and better reusability compared to previously reported Fe_3_O_4_ composites or hBN-based materials. Therefore, the IOhBN system represents an advanced class of magnetic nanosorbents that effectively combine magnetic and adsorptive functionalities within a single, stable, and green material design.

### 3.11. Greenness assessment of the IOhBN/mSPE method

**Figure 4:**
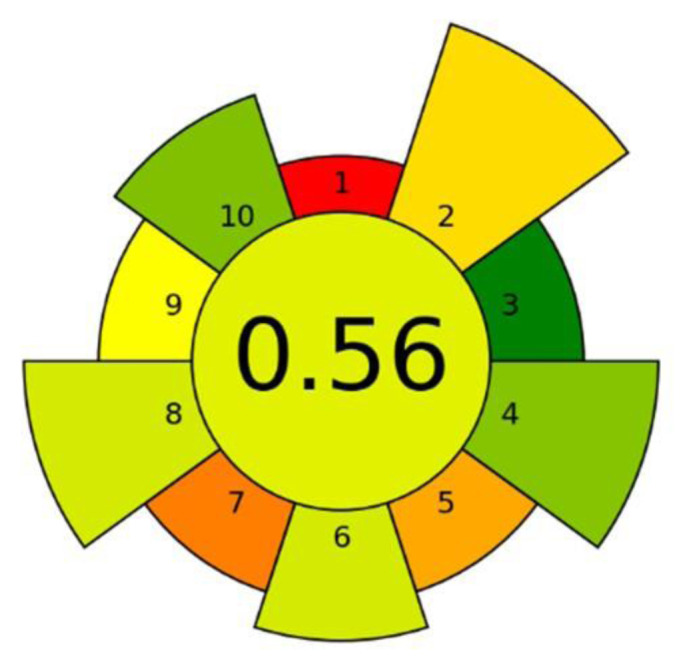
Greenness assessment pictograph of the developed IOhBN/mSPE procedure based on the 10 principles of the AGREEprep metric, resulting in a final score of 0.56.

To holistically assess the environmental sustainability of the proposed IOhBN/mSPE procedure, the analytical greenness metric for sample preparation (AGREEprep) technique, was employed [46]. The analysis yielded an overall score of 0.56, which is depicted in the resulting pictograph (Figure 4). This score indicates an acceptable and moderate level of greenness for the sample preparation method. The primary factors detracting from a higher score are inherent to the procedure’s design. Notably, the method is ex situ, requiring sample collection and off-site laboratory preparation, which inherently results in limitations compared to in situ techniques. Furthermore, the use of hazardous reagents, specifically the 3.0 mol L^−1^ of HNO_3_ as an eluent and NH_4_OH during sorbent synthesis, negatively impacts the score. The manual nature of the procedure, relying on vortexing and manual magnetic decantation, also limits the score in the automation and integration criteria. Despite these drawbacks, the score of 0.56 is well supported by several positive attributes. The method achieves high scores for its minimal material consumption, utilizing only 15 mg of sorbent and 0.5 mL of eluent per analysis, which directly corresponds to minimal waste generation. The procedure’s rapidity (5-min extraction cycle) is a significant green advantage. Therefore, while the AGREEprep analysis highlights areas for improvement, particularly regarding reagent selection and automation, it confirms that the IOhBN/mSPE method holds a respectable position in terms of environmental friendliness, primarily driven by its high efficiency and minimization of material and energy inputs.

## 4 Conclusion

A novel magnetic nanosorbent based on IOhBN was successfully synthesized through a one-step, surfactant-free coprecipitation method and applied for mSPE of Co(II) ions in water and food samples. The IOhBN composite exhibited high stability, strong magnetic response, and efficient adsorption capacity due to the synergistic combination of Fe_3_O_4_ and hBN components. Under optimized conditions (pH 7.0, 15 mg of sorbent, 0.5 mL of 3.0 mol L^−1^ HNO_3_, and 5-min extraction time), the method achieved an LOD of 0.67 μg L^−1^, an RSD of 4.2%, and an EF of 20. The procedure was validated using CRMs and successfully applied to various real samples, confirming its reliability and practical applicability. Compared to existing Fe_3_O_4_- and BN-based sorbents, the IOhBN system offers comparable sensitivity with the added advantages of being faster and environmentally benign, as evidenced by its Eco-Scale score of 82. However, certain limitations remain, such as the selectivity toward Co(II) could be further improved in the presence of competing ions at very high concentrations, and large-scale synthesis reproducibility should be studied in future work.

Future research will focus on surface functionalization of IOhBN with chelating ligands to enhance selectivity, as well as extending the application of this green magnetic sorbent to other trace metal ions and environmental monitoring systems. Overall, the present study provides a promising and sustainable platform for rapid, cost-effective, and environmentally friendly preconcentration of trace metals.

## Figures and Tables

**Figure 2A:**
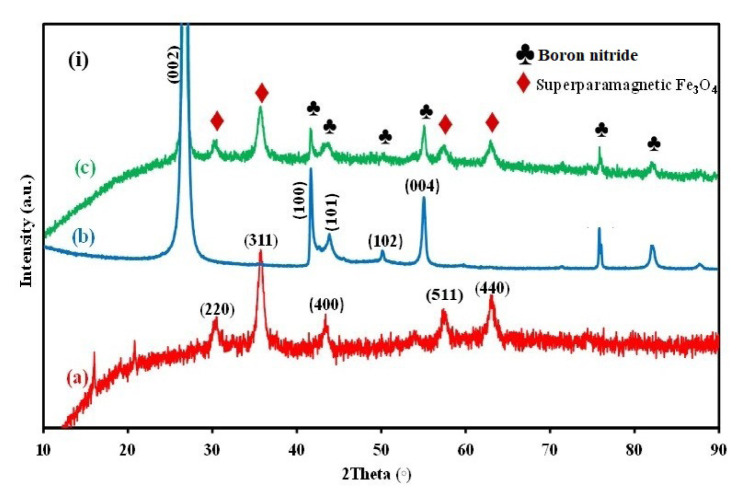
XRD spectra of the IO (a), hBN (b), and IOhbN (c) materials.

**Figure 2B:**
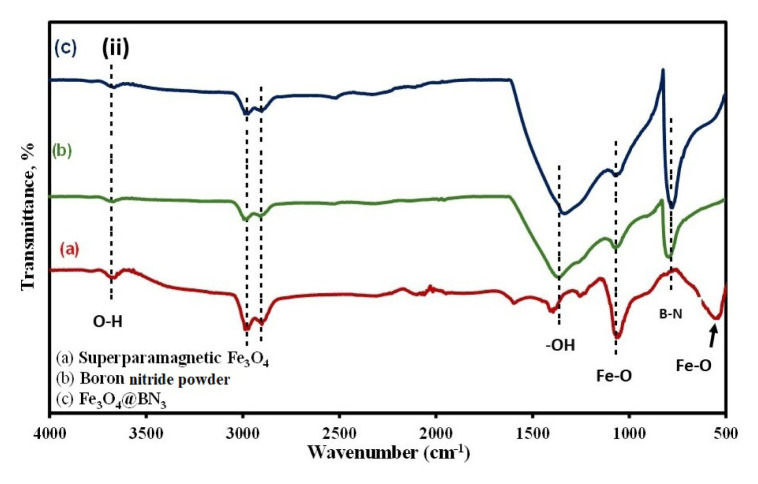
FT-IR spectra of the IO (a), hBN (b), and IOhbN (c) materials.

**Figure 2C:**
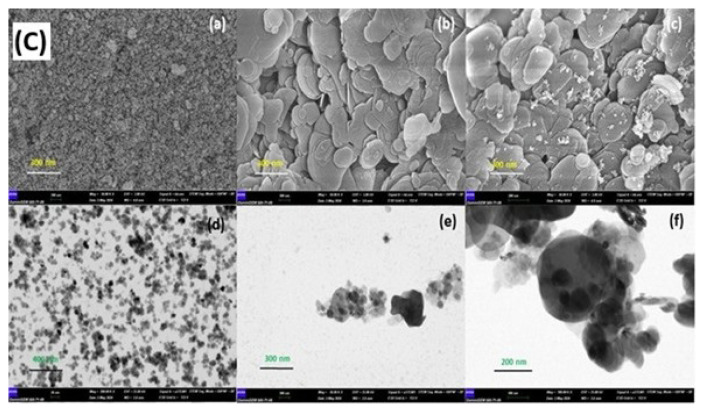
FE-SEM images of IO (a), hBN (b) and IOhbN (c), and SEM images of IO (d), hBN (e), and IOhbN (f).

**Figure 2D:**
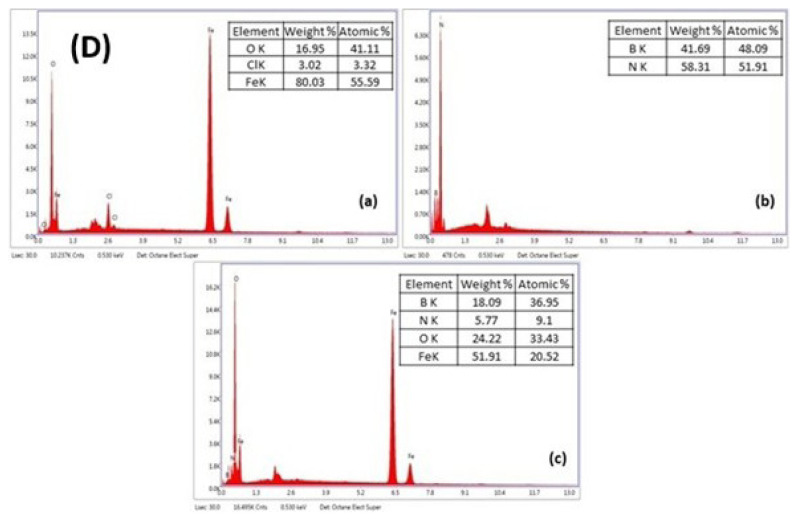
EDX results of IO (a), hBN (b), and IOhbN (c).

**Table 1:** Effect of diverse ions on the recovery of Co(II) (40 μg L^−1^ Co(II), pH 7.0, 15 mg adsorbent, 2 min adsorption, 3 min desorption, 10 mL sample volume, 0.5 mL eluent (3.0 mol L^−1^ HNO_3_)), (N = 3).

Ions / added as	Concentration, mg L^−1^	Recovery, % *
Pb^2+^/ Pb(NO_3_)_2_	2500	97±6
K^+^ / *KCl*	2500	96±5
Cu^2+^ / Cu*(NO_3_)_2_.6H_2_O*	5	99±11
Cr^3+^ / Cr(NO_3_)_3_.9H2O	20	93±6
CO_3_^2−^ / *Na_2_CO_3_*	500	94±6
Mg^2+^ / *MgCl_2_.6H_2_O*	250	96±11
SO_4_^2−^ / *Na_2_SO_4_*	2500	96±7
Ni^2+^ / *Ni(NO_3_)_2_.6H_2_O*	10	89±4
Na^+^/*NaNO_3_*	2500	91±2
Cl^−^ / *KCl*	2500	96±5
NO_3_^−^ / *NaNO_3_*	500	91±2

* mean ± standard deviation.

**Table 2:** Application of IOhBN/mSPE method on CRMs (pH 7.0, 15 mg of adsorbent, 2 min of adsorption, 3 min of desorption, 10-mL sample volume, 0.5 mL of eluent (3.0 mol L^−1^ of HNO_3_)), (N = 3).

CRMs	Certified	Found	Recovery, %*
**Onion (μg g^−1^)**	0.59 ± 0.04	0.54 ± 0.04	92
**Spinach leaves (μg g^−1^)**	0.393 ± 0.03	0.365 ± 0.02	93
**Estuarine water (nmol kg^−1^)**	0.99 ± 0.26	1.04 ± 0.07	105

* Mean ± standard deviation, spinach leaves: NIST 1570a, onion: NCS ZC 73033, estuarine water: BRC 505.

**Table 3:** Spiked Co(II) in food samples and Co(II) levels in selected food samples (pH 7.0, 15 mg of adsorbent, 2 min of adsorption, 3 min of desorption, 10-mL sample volume, 0.5 mL of eluent (3.0 mol L^−1^ of HNO_3_), (N = 3).

Sample	Added, μg g^−1^	Found, μg g^−1^	Recovery, %
Spinach leaves	0	0.71 ± 0.05	-
	1	1.76 ± 0.09	105
	2.5	3.23 ± 0.12	101
Onion	0	0.36 ± 0.02	-
	1	1.31 ± 0.09	95
	2.5	2.81 ± 0.14	98
Coffee	0	0.43 ± 0.02	-
	1	1.47 ± 0.11	104
	2.5	2.95 ± 0.19	101
Tuna fish	0	0.029 ± 0.001	-
	0.05	0.083 ± 0.002	108
	0.1	0.125 ± 0.009	96

* Mean ± standard deviation.

**Table 4:** Application of IOhBN/mSPE method for Co(II) determination in tap water and wastewater samples (pH 7.0, 15 mg of adsorbent, 2 min of adsorption, 3 min of desorption, 10-mL sample volume, 0.5 mL of eluent (3.0 mol L^−1^ HNO_3_)), (N = 3).

Sample	Added, μg mL^−1^	Found, μg mL^−1^	Recovery, %
Tap Water-1	0	ND	-
	0,05	0.054 ± 0.001	108
	0.1	0.107 ± 0.003	107
Tap Water-2	0	ND	-
	0.05	0.046 ± 0.003	92
	0.1	0.090 ± 0.005	90
Wastewater-1	0	0.092 ± 0.005	-
	0.05	0.139 ± 0.009	94
	0.1	0.198 ± 0.012	106
Wastewater-2	0	0.081 ± 0.003	-
	0.05	0.128 ± 0.008	94
	0.1	0.171 ± 0.009	90

ND: No detection;

* mean ± standard deviation.

**Table 5:** Comparison of the suggested IOhBN/mSPE technique with other preconcentration studies of Co(II).

Sorbent	Extraction technique	Detection technique	Matrix	LOD, μg L^−1^	PF	%RSD	Reference
Fe_3_O_4_@coPPy-PTH nanocomposite	mSPE	MIS-FAAS	Food and water	0.17	25	1.5	[47]
Functionalized biopolyamide	NH-IS-SPE	μS-FAAS	Food and water	2.95	200	3.5	[48]
Grape stalk-based active carbon	SPE	HR- CS FAAS	Food and water	0.27	150	2.3	[49]
Polyvinyl chloride modified by 3-(2-thiazolylazo)-2,6-diaminopyridine	SPE	FAAS	Food and water	1.3	40	1.8	[50]
Magnetic carboxyl-modified nanodiamond	SPE	FAAS	Water	1.73	20	0.7	[51]
IOhBN	mSPE	HR-CS FAAS	Food and water	0.67	20	4.2	This work

NH-IS-SPE: Needle hub in-syringe solid phase extraction, MIS-FAAS/μS-FAAS: microsample injection system-flame atomic absorption spectrometry, LOD: limit of detection, PF: preconcentration factor, RSD: relative standard deviation.
